# Predictors for post-traumatic hip osteoarthritis in patients with transverse acetabular fractures following open reduction internal fixation: a minimum of 2 years’ follow-up multicenter study

**DOI:** 10.1186/s12891-023-06945-2

**Published:** 2023-10-13

**Authors:** Junran Li, Lin Jin, Chuanjie Chen, Jingxiu Zhai, Ligeng Li, Zhiyong Hou

**Affiliations:** 1 Department of Orthopedic Surgery, Second Hospital of Tangshan, Tangshan, 063000 Hebei P.R. China; 2 Institute of Trauma Surgery, Second Hospital of Tangshan, Tangshan, 063000 Hebei P.R. China; 3 Department of Orthopaedic Surgery, Third Hospital of Hebei Medical University, Shijiazhuang, 050051 Hebei P.R. China; 4 Department of Orthopedic Surgery, Chengde Central Hospital, Chengde, 067000 Hebei P.R. China

**Keywords:** Transverse acetabular fracture, Open reduction internal fixation, Post-traumatic osteoarthritis, Predictors

## Abstract

**Background:**

The predictors of post-traumatic osteoarthritis (PTOA) in patients with transverse acetabular fractures (TAFs) following open reduction internal fixation (ORIF) remain unclear. This study aimed to investigate the risk factors for PTOA in TAFs after ORIF.

**Methods:**

Data of TAF patients receiving ORIF were collected from January 2012 and February 2021. Patients suffered PTOA were classified as the osteoarthritis group (OG), while those without PTOA were classified as the non- osteoarthritis group (NG) with a minimum follow-up of 2 years. PTOA was diagnosed according to Tönnis OA classification during the period of follow-up. Univariate analysis, logistic regression analysis, and receiver operating characteristic (ROC) curve analyses were used to evaluate demographics, injury-related characteristics, perioperative and post-discharge information.

**Results:**

Three hundred and eleven TAF patients were analyzed in this study, including 261 males and 50 females, with a mean age of 40.4 years (range 18 to 64 years). The incidence of PTOA was 29.6% (92 of 311) during the mean follow-up of 36.8 months (range 24 to 70 months). Several factors of PTOA were found using univariate analysis, including transverse fracture associated with posterior wall acetabular fracture (TPW-AF, *p* = 0.002), acetabular roof fracture (ARF, *p* = 0.001), femoral head lesion (FHL, *p* = 0.016), longer time from injury to surgery (TIS, *p*＜0.001) and physical work after surgery (PWAS, *p*＜0.001). Logistic regression analysis showed that TPW-AF (*p* = 0.007, OR = 2.610, 95%CI: 1.302–5.232), ARF (*p* = 0.001, OR = 2.887, 95%CI: 1.512–5.512), FHL (*p* = 0.005, OR = 2.302, 95%CI: 1.283–4.131), TIS (*p*<0.0001, OR = 1.294, 95%CI: 1.192–1.405) and PWAS (*p*<0.0001, 3.198, 95%CI: 1.765–5.797) were independent risk factors of PTOA. Furthermore, ROC curve analysis indicated 11.5 days as the cut-off values to predict PTOA.

**Conclusions:**

Our findings identified that TPW-AF, ARF, FHL, TIS and PWAS were independent risk factors for PTOA in patients with TAFs following ORIF. It can help orthopedic surgeons to take early individualized interventions to reduce its incidence.

## Introduction

As the rapid development of society, high-energy acetabular injuries caused by industry and transportation have increased annually [1]. Surgical treatment for acetabular fracture has always remained challenging because of complex anatomical relationships to its surrounding tissues and organs [2]. Some studies have reported that the incidence of postoperative secondary osteoarthritis after surgical reduction and fixation of acetabular fracture can be as high as 20-25%, and most patients suffered from painful hip and limited activity, which seriously affected quality of life, so much so that some patients had to require reoperation [3–5]. Therefore, it appears to be particularly significant to identify the risk factors affecting the development of post-traumatic osteoarthritis (PTOA) after operative management of acetabular fractures.

Currently, the most widely applied to categorize acetabular fractures is the Letournel classification [6–8]. Based on Letournel’s 2-column theory, discontinuities of the iliopectineal lines or the ilioischial lines on X-ray film were defined as anterior column or posterior column fractures, respectively [6, 7, 9]. The pure transverse acetabular fracture (PTAF) and transverse fracture associated with posterior wall acetabular fracture (TPW-AF) in Letournel classification are representative of injury patterns that simultaneously rupture both anterior and posterior columns. The injury mechanisms of these two fracture types are quite similar, and innominate bone is separated into both the upper and the lower parts (ilium and puboischium) by the main transverse fracture line [10]. However, in clinical practice, intraoperative reduction for fracture fragments would be difficult considering that the lesions of transverse type were simultaneously involved both two columns [11]. As a consequence, postoperative function of transverse acetabular fractures (TAFs) is regarded to be worse than those of other types [12–14].

Previous studies [12, 15, 16] reported that several risk factors for the development of PTOA in patients with acetabular fractures after treating by open reduction internal fixation (ORIF), such as demographic variables, injury-related characteristics and perioperative information [5, 17, 18]. To our knowledge, few studies have focused on the predictors of PTOA following TAFs after ORIF. The propose of this study was to identify the risk factors of PTOA for TAF patients who treated by ORIF.

## Materials and methods

### Study design and patients

After institutional review board approval of the participating institutions, this retrospective study collected the data of TAFs patients, was performed in electronic databases of three level I trauma centers, between January 2012 and February 2021. A total of nineteen orthopaedic surgeons performed these operations by using plate fixation in treating displaced TAFs. The inclusion criteria were as follows:(a) patients aged between 18 and 65 years; (b) patients diagnosed with TAFs (PTAFs or TPW-AFs); (c) patients receiving ORIF; and (d) time from injury to surgery less than 21 days. The exclusion criteria were as follows: (a) pathologic fractures; (b) open pelvic or acetabular fractures; (c) preexisting ipsilateral hip diseases or developmental deformities; and (d) less than 2-year follow-up, or loss of follow-up. The informed consent forms were obtained from all participants.

### Data collection and assessment

The demographics, injury-related characteristics, perioperative and post-discharge information were reviewed in this study. The demographic characteristics consisted of age, gender, residential location, smoking, alcohol, comorbidities (hypertension, diabetes mellitus, cerebral infarction and heart disease), American society of anesthesiologists (ASA, grade I-II and grade III-IV), body mass index (BMI,＜18.5, 18.5–23.9, 24-27.9, 28-29.9, and ≥ 30 kg/m^2^). The injury-related characteristics were acetabular fracture type (Letournel classification), side, mechanism of injury (motor vehicle collision, fall from height and others), and concurrent hip dislocation, acetabular roof fracture (ARF), femoral head lesion (FHL), pelvic ring injury (PRI), femoral neck fracture (FNF), femoral intertrochanteric fracture (FIF). The perioperative and post-discharge information included time from injury to surgery (TIS), intraoperative blood loss (IBL), duration of operation, surgical approach (anterior intrapelvic, Kocher-Langenbeck and both two approaches), quality of reduction (satisfactory and unsatisfactory reduction), and physical work after surgery (PWAS). Quality of reduction was evaluated by postoperative radiograph based on the grading criteria described by Matta [19]. The anatomic, imperfect or poor reduction was stratified as the displacement < 1 mm, 2–3 mm or > 3 mm, respectively. Anatomic and imperfect reductions were regarded as satisfactory reductions in this study.

All patients were categorized and diagnosed with TAFs according to both Letournel and AO/OTA classifications [6, 7, 20] by using radiographs and computed tomography (CT) images. Preoperative imaging features were evaluated by two senior orthopedists who specialized in pelvic and acetabular trauma surgery with over 10 years of experience. Postoperative diagnose for PTOA was confirmed by two trained orthopedists by referring to Tönnis OA classification [21] with follow-up radiographs. The results of imaging evaluation and other information were uniformly recorded by the person who was not participate the evaluative work in the same orthopedic medical center.

Patients suffered post-traumatic hip osteoarthritis were classified as the osteoarthritis group (OG), while those without osteoarthritis were classified as the non- osteoarthritis group (NG) with a minimum follow-up of 2 years.

### Statistical analysis

Statistical analyses were conducted in SPSS statistics version 25.0 software (IBM, Armonk, NY, USA). The p value ＜0.05 was regarded as statistical significance in this study. Continuous variables were presented as the means ± standard deviations, whereas categorical variables were described as numbers and percentages (%). Independent samples Student’s t tests were used for continuous variables with a normal distribution; otherwise, the Mann-Whitney U test was used to perform statistical analysis between groups. Differences in categorical variables were determined using the chi-squared test or Fisher`s exact test. Moreover, to identify independent risk factors for PTOA after ORIF in patients with TAFs, binary logistic regression analysis was used to detect independent predictors of PTOA. In addition, receiver operating characteristic (ROC) curves were utilized to identify the optimal cut-off values for continuous variables, namely when the Youden index (sensitivity + specificity − 1) was maximum. The area under the ROC curve (AUC), which ranged from 0 to 100%, with more area presenting better ability, was applied to determine the diagnostic capability. Odds ratios (ORs) with 95% confidence intervals (CIs) were used to indicate the correlation strength.

## Results

**Fig. 1:**
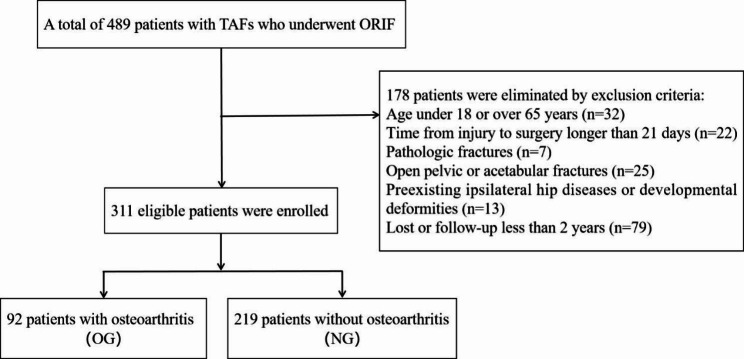
Flow diagram of included patients

In total, 427 patients with TAFs were enrolled during the study interval. On the basis of the exclusion criteria, 311 TAF patients were analyzed in this study, including 261 males and 50 females, with a mean age of 40.4 years (range 18 to 64 years). During the mean follow-up of 36.8 months (range 24 to 70 months), there were 92 developed a post-traumatic hip osteoarthritis in patients with TAFs, all of whom underwent ORIF, while 219 were without PTOA (Fig. 1). Among these patients, the incidence of PTOA after ORIF was 29.6%.

As shown in Table 1, we found there were statistically significant differences between the NG and OG in terms of TPW-AF (*p* = 0.002), ARF (*p* = 0.001) and FHL (*p* = 0.016). This table indicated that patients in the OG were more likely to present TPW-AF, ARF or FHL than those in NG. However, there were no significant differences between the two groups in terms of age, gender, residential location, smoking, alcohol, comorbidities, ASA, BMI, side, mechanism of injury, hip dislocation, PRI, FNF and FIF (*p*＞0.05).

**Table 1:** Comparison of demographics and injury-related characteristics between the two groups

	Non-osteoarthritis group (n = 219)	Osteoarthritis group (n = 92)	*p* value
**Age (years)**	40.0 ± 12.0	41.6 ± 11.4	0.267
**Gender (male), n (%)**	182 (83.1)	79 (85.9)	0.545
**Residential location (urban), n (%)**	93 (42.5)	31 (33.7)	0.149
**Smoking, n (%)**	75 (34.2)	26 (28.3)	0.304
**Alcohol, n (%)**	48 (21.9)	17 (18.5)	0.496
**Comorbidities**			
Hypertension, n (%)	16 (7.3)	5 (5.4)	0.548
Diabetes mellitus, n (%)	5 (2.3)	4 (4.3)	0.458
Cerebral infarction, n (%)	1 (0.5)	2 (2.2)	0.210
Heart disease, n (%)	9 (4.1)	5 (5.4)	0.607
**ASA, n (%)**			0.318
I-II	171 (78.1)	67 (72.8)	
III-IV	48 (21.9)	25 (27.2)	
**BMI, n (%)**			0.275
< 18.5	17 (7.8)	5 (5.4)	
18.5–23.9	36 (16.4)	15 (16.3)	
24–27.9	89 (40.6)	30 (32.6)	
28–29.9	51 (23.3)	23 (25.0)	
≥ 30	26 (11.9)	19 (20.7)	
**Fracture type (Letournel), n (%)**			0.002
Pure transverse fracture	77 (35.2)	16 (17.4)	
Transverse associated with posterior wall fracture	142 (64.8)	76 (82.6)	
**Side (left), n (%)**	122 (55.7)	49 (53.3)	0.692
**Mechanism of injury, n (%)**			0.287
Motor vehicle collision	130 (59.4)	55 (59.8)	
Fall from height	44 (20.1)	24 (26.1)	
Others	45 (20.5)	13 (14.1)	
**Acetabular fracture plus**:			
Hip dislocation, n (%)	49 (22.4)	22 (23.9)	0.768
Acetabular roof fracture, n (%)	120 (54.8)	69 (75.0)	0.001
Femoral head lesion, n (%)	73 (33.3)	44 (47.8)	0.016
Pelvic ring injury, n (%)	121 (55.3)	56 (60.9)	0.361
Femoral neck fracture, n (%)	21 (9.6)	10 (10.9)	0.731
Femoral intertrochanteric fracture, n (%)	13 (5.9)	7 (7.6)	0.583

*ASA* American Society of Anesthesiologists, *BMI* body mass index

The comparison of perioperative and post-discharge information in patients with or without PTOA are presented in Table 2. We found that the OG had significantly longer TIS (*p*＜0.001) than NG. Furthermore, PWAS (*p*＜0.001) were also found to be associated with the risk of PTOA after ORIF. Additionally, there were no significant differences in other perioperative data between these two groups (*p*＞0.05).

**Table 2:** Comparison of perioperative and post-discharge information between the two groups

	Non-osteoarthritis group (n = 219)	Osteoarthritis group (n = 92)	*p* value
**Time from injury to surgery (days)**	8.4 ± 3.1	11.2 ± 4.0	＜0.001
**Intraoperative blood loss (ml)**	960.3 ± 339.2	936.4 ± 285.4	0.766
**Duration of operation (min)**	171.7 ± 36.0	165.6 ± 31.1	0.158
**Surgical approach, n (%)**			0.262
Anterior intrapelvic	19 (8.7)	7 (7.6)	
Kocher-Langenbeck	147 (67.1)	70 (76.1)	
Both	53 (24.2)	15 (16.3)	
**Quality of reduction (satisfactory reduction), n (%)**	156 (71.2)	69 (75.0)	0.498
**Physical work after surgery, n (%)**	53 (24.2)	42 (45.7)	＜0.001

The logistic regression analysis presented that TPW-AF [*p* = 0.007, OR = 2.610, 95%CI (1.302, 5.232)], ARF [*p* = 0.001, OR = 2.887, 95%CI (1.512, 5.512)], FHL [*p* = 0.005, OR = 2.302, 95%CI (1.283, 4.131)], TIS [*p*＜0.0001, OR = 1.294, 95%CI (1.192, 1.405)] and PWAS [*p*＜0.0001, OR = 3.198, 95%CI (1.765, 5.797)] were independent risk factors for PTOA (Table 3). Moreover, no protective factors that impact the incidence of PTOA in these patients with TAFs after ORIF were observed in this study (Table 3).

**Fig. 2:**
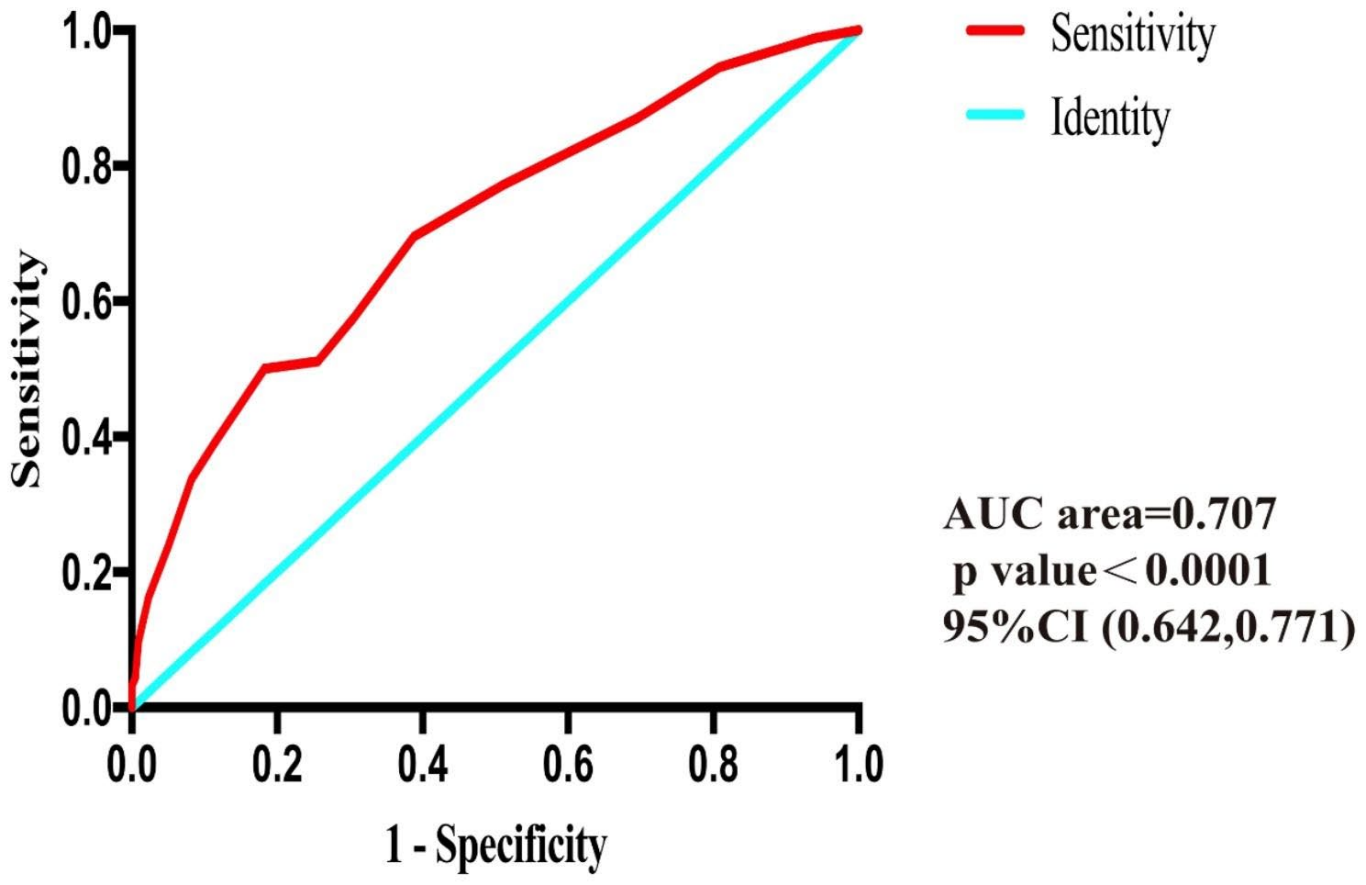
Risk factors of post-traumatic osteoarthritis following transverse acetabular fractures in receiver operating characteristic curve analysis

The optimum cut-off value of TIS to determine the independent predictor of post-traumatic hip osteoarthritis following TAFs after ORIF which we analyzed was 11.5 days by the ROC curve [*p* < 0.0001, AUC area = 0.707, 95%CI (0.642, 0.771)] (Fig. 2).

**Table 3:** Logistic regression analysis of variables associated with post-traumatic hip osteoarthritis

Variables	B	S.E.	Wald	OR	95%CI	*p* value
TPW-AF	0.959	0.355	7.312	2.610	1.302–5.232 1.512–5.512 1.283–4.131 1.192–1.405 1.765–5.797	0.007
ARF	1.060	0.330	10.326	2.887		0.001
FHL	0.834	0.298	0.298	2.302		0.005
TIS	0.258	0.042	37.558	1.294		< 0.0001
PWAS	1.163	0.303	0.303	3.198		< 0.0001

*OR* odds radio, *CI* confidence interval, *TPW-AF* transverse associated with posterior wall fracture, *ARF* acetabular roof fracture, *FHL* femoral head lesion, *TIS* time from injury to surgery, *PWAS* physical work after surgery

## Discussion

TAFs commonly occur related to severe trauma and require surgical treatment [22]. However, it often results in some postoperative complications, such as heterotopic ossification, deep venous thrombosis, infection, or secondary hip osteoarthritis [4]. Kelly [13] retrospectively reviewed 8389 patients with acetabular fractures after ORIF and found that PTOA was the most common complication during the follow-up. PTOA, one of the tough postoperative complications, is a clinical condition caused by trauma or other factors that leads to persistent pain, limited activity, and even disability [3–5]. To date, total hip arthroplasty is a popular and effective treatment for irreversible disorders of the hip, which can enhance the moving ability of lower limbs sooner [23]. Even so, early identification and intervention of its risk factors can be helpful to reduce the incidence of re-operation and improve patients` quality of life significantly. In recent years, few studies have concerned with risk factors that might have affected the prognosis of patients with TAFs who had experienced an ORIF. To the best of our knowledge, this is the first study to analyze demographics, injury-related characteristics, perioperative and post-discharge information associated with the occurrence of PTOA for TAF patients after ORIF.

In our research, we retrospectively reviewed patients with TAFs following ORIF and found that the rate of PTOA was 29.4% (115 of 391) within over 2 years of clinical follow-up. The results of logistic regression analysis showed that TPW-AF, ARF, FHL, TIS and PWAS were independent predictors for PTOA. ROC curve analysis indicated that the cut-off values for TIS to predict PTOA was 11.5 days. Furthermore, the highest diagnostic accuracy was presented when TPW-AF, ARF, FHL, TIS and PWAS were all taken together.

Our study found an increased incidence of PTOA in patients with TAFs complicated with posterior wall fractures (i.e. TPW-AFs). Letournel classification, the most common method utilized to categorize acetabular fractures, divides TAFs and TPW-AFs into elementary fracture type and associated fracture type based on the geometric form of the fracture line, respectively [6, 7]. In general, surgical difficulties and postoperative outcomes of the elementary acetabular fractures are considered to be better than those of associated fractures [24]. Kreder [25] found that the presence of poor functional outcomes resulted from complex acetabular fractures associated posterior wall fractures. Rollmann [5] also has reported that involvement of the posterior wall was an independent predictor of secondary hip osteoarthritis, which was consistent with our findings. It can also be confirmed theoretically that the complex acetabular fractures involving the posterior wall account for a poor prognosis due to the high rate of comminution or bone defects, which leads to hip instability, eventually, generating secondary osteoarthritis [24]. Therefore, at the time of treating complex acetabular fractures involved posterior wall fractures, surgeons should highlight the need for accuracy of reduction and give high priority in clinical practice.

In our study, patients with ARF or FHL had an increased incidence of PTOA. We also observed that ARF and FHL increased the prevalence of PTOA by roughly 1.9-fold and 2.0-fold, respectively, compared to those without these injuries. It is well known that weight-bearing area broken is a relatively serious injury pattern that is prone to change the biomechanical relationship between femoral head and acetabulum [17]. With abnormal load on the surface of hip joint, the development of PTOA would be aggravated in walking, standing, squatting, or sitting position [26]. Lubovsky [27] reported that patients who sustained acetabular roof fractures usually had poor functional outcomes following ORIF. In addition, previous studies reported that acetabular fractures associated osteochondral or chondral lesions of femoral head can also exacerbate articular incongruity, leading to the acceleration of degenerative changes to the hip joint [19, 28, 29]. Rollmann and Clarke-Jenssen all demonstrated that FHL represented an independent risk factor for PTOA [4, 5], which was consistent with our study. As a result, while for the most part ARF or FHL are unavoidable, performing a detailed assessment of the injury, making an appropriate surgical planning, optimizing rehabilitation of joint function, and improving the level of care are crucial measures to reduce the occurrence of PTOA.

Clinically, it is widely known that delay in fixation of fracture negatively affects reduction quality and corresponding functional outcomes, especially for the acetabulum [30]. The duration of TIS was significantly longer in the osteoarthritis group compared with the non-osteoarthritis group in this study. Previous researches have shown that surgical intervention of acetabular fractures at early date can be associated with higher anatomical reduction rates, have beneficial effect on decreasing the occurrence of secondary hip osteoarthritis [24, 31]. It also has been demonstrated that the duration of TIS was as a significant risk factor, which was in line with our findings. Periacetabular hematoma, fibrous scars, osteo-callus and adhesions of soft tissues were presented in patients with longer TIS and increasing difficulty during surgical reduction [32]. These factors could result in reestablished an unstable and incongruent joint and developed articular degeneration, which cause PTOA [33, 34]. Specifically, acetabular surgeons should minimize the interval between injury to operation as soon as possible to decrease the probability of developing PTOA after patients with resuscitated and stabilized situation.

In addition to the relevant factors mentioned above, PWAS also had an effect on the occurrence of PTOA in patients with TAFs after treating by ORIF. We observed that patients with PWAS had a 1.9-fold increased incidence of PTOA compared to those without PWAS. This result may be due to the fact that the excessive pressure during movement of hip joint is directed to the fracture line and leads to displacement, which increases the risk of PTOA [35]. It could also be caused by the over-load on the articular surface contributing to stress concentration, resulting in lesions of cartilage and further degeneration [36]. However, intermittent and low shear stress has advantages on the regeneration to the collagen fibers of articular cartilage [36]. Phruetthiphat [37] has identified that postoperative activity restriction in acetabular fracture patients is essential for improving joint function, and appropriate low-intensity exercise is beneficial for postoperative recovery. Therefore, for patients who take physical labor as their profession before injury, it is recommended to change their work and lifestyle with less load after surgery, gradually increasing the weight bearing intensity of the joint, and avoiding secondary injury to the hip joint caused by excessive pressure, thereby effectively reducing the risk of regeneration.

### Limitations

Although this was the first study to specifically investigate PTOA risk factors in patients with TAFs after ORIF, several limitations should be noted. First, since this research was retrospective, certain potential variables that may be connected to PTOA, such as bone mineral density and functional outcome, were only partially detected. Second, other unknown factors including surgeon’s understanding of the fracture, habits, and experience may also have affected, which were not considered in the assessment. Finally, due to relatively rare number of TAF patients, a multi-center study was performed to enlarge sample sizes. However, the differences of data collection in each trauma center must be taken into consideration when interpreting our results.

## Conclusions

Although ORIF was the routine treatment for TAFs, the incidence of postoperative trauma-related osteoarthritis was still high; however, its risk factors remain unclear. In this study, we found that TPW-AF, ARF, FHL, TIS and PWAS were independent risk factors for PTOA in patients with TAFs treated by ORIF. It can help clinical practitioners to take early individualized interventions to reduce its incidence.

## Data Availability

The datasets used and analyzed during the current study are available from the corresponding author upon reasonable request.

## Abbreviations

PTOA: Post-traumatic osteoarthritis
TAFs: Transverse acetabular fractures
PTAF: Pure transverse acetabular fracture
TPW-AF: Transverse associated with posterior wall acetabular fracture
ORIF: Open reduction internal fixation
BMI: Body mass index
ASA: American society of anesthesiologists
ARF: Acetabular roof fracture
FHL: Femoral head lesion
PRI: Pelvic ring injury
FNF: Femoral neck fracture
FIF: Femoral intertrochanteric fracture
TIS: Time from injury to surgery
IBL: Intraoperative blood loss
PWAS: Physical work after surgery
CT: Computed tomography
ROC: Receiver operating characteristic
AUC: Area under the curve
